# Supplementary material for: Forecasting the dynamics of a complex microbial community using integrated meta-omics
Source: Nat Ecol Evol. 2023 Nov 13;8(1):32–44. doi: 10.1038/s41559-023-02241-3 (PMC10781640; doi:10.1038/s41559-023-02241-3)
Supplement: Supplementary file 1 — Supplementary discussion and legends for Supplementary Tables 1–7. [file 41559_2023_2241_MOESM1_ESM.pdf]

nature ecology & evolution

Article https://doi.org/10.1038/s41559-023-02241-3

Forecasting the dynamics of a complex microbial community using integrated meta-omics

In the format provided by the authors and unedited

Supplementary Information

Time series analysis of biological data

There exist several categories of time-series analysis. These are based on: i) previous knowledge (such as curve fitting[1] and classification[2,3], ii) subsetting (e.g. segmentation[4]), iii) clustering (e.g. based on various metrics such as Euclidean Distance[5] or Dynamic Time Warping[6]) iv) prediction (such as forecasting[7] and intervention analysis[8]), and v) decomposition (e.g. Singular Value Decomposition - SVD[9]). The prediction of future states of ecological communities and their interplay with the environment have been successfully tackled in the case of available interaction models and/or limited number of species[10,11]. However, predictions of microbial metabolic behaviour are rendered challenging for naturally occurring microbial ecosystems as well as industrially-relevant ones, such as in BWWTPs. In this context, metagenomics (MG)[12,13], metatranscriptomics (MT)[14] and metaproteomics (MP)[15] enable the establishment of meta-omic sample-specific reference databases that simultaneously resolve both compositional and functional aspects of the system.

Ecological hypothesis of clique C2

The second clique, C2, includes S9, S4 and S7 leading to S8. Both S4 and S8 represent oscillatory “perturbations" (Figure 2b, **Supplementary Figure 7d**). Whilst S4 is increasing in amplitude, S8 is decreasing. Interestingly, out of the four only S8 has an autoregressive component and S7 is missing any seasonal signal (**Figure 2b**). The nitrogen-associated S9 has a simple dependency on NH_4 (**Figure 2b**) and indeed influences positively the family Nitrosomonadaceae (**Supplementary Figure 10**). S7 is weakly influenced by seasonality and has a relatively strong intercept (**Figure 2b**) but is affected by both pH and NH_4. The bacterial taxonomic contributions to S7 show a mixed response of the transcriptome whereby the only positive MG association is with the viral family Mimiviridae. It is possible that S7 encodes fluctuations in the parameters and the immediate response of the microbiome (through RNA), without a defined overarching pattern. The pair S4 and S8 are however more intriguing, because of the counterintuitive idea that an escalating perturbation could contribute to the resolution of another perturbation. S4 is explained solely by seasonal components, whilst S8 also includes pH effects from both the sampling site and the inflow, even if with opposite effects (**Figure 2b**). The signal S4 is -in general- negatively associated with gene expression and protein levels, however it is positively impacted by the level of the putative predator Nannocystaceae[16]. The functional

associations of S8 include a negative one for porphyrin and chlorophyll and positive ones for glycerophospholipids and simple sugars, hinting at a switch between autotrophic and medium-dependent metabolisms in the foam community (**Supplementary Figure 11**). This seems to suggest that an interplay between the predation by the family Nannocystaceae, supported by parameter fluctuations in pH and NH_4 might lead to further general instability in the RNA expression of the microbiome. Even more curious is how the exacerbation of the amplitude of S4 might drive the stabilisation of S8, according to the idea that higher predation levels have been linked to the stability of ecosystems[17]. Moreover, S4 might play a role in the cyclical ecology of the system beyond the environmental variables (indeed we did not find any associated with it). On the other hand S5 shows no link to seasonality but is positively influenced by aeration. Among the taxa that contribute to it the most, there are families known to be involved in the bulking process such as Gordoniaceae and Zoogloeaceae. This points to a putative connection between the bulking process and the aeration, which is beyond seasonal effects.

Time-independent Fatty Acid and Triacylglycerol accumulation

For a LAO community, the biosynthesis of Triacylglycerol (TG) and Fatty Acids (FA) are crucial steps[18] involving multiple enzyme classes and with several entry points (**Figure 3a**). The abundant and expressed classes cover the circuit going from Acyl-Phosphate (Acyl-P) to fatty acid (FA) as shown in **Figure 3a**, however none of the enzymes' quantities are in the top/bottom 5% of the loadings for the time-dependent EGs. It looks, in general, that the accumulation of TG and FA is time-independent. This is consistent with the observation that functions are mostly conserved in a BWWTP[19]. Interestingly K22848 is mostly encoded and expressed by the family Moraxella which is one of the two dominant families in the system **(Figure 3b**). Together with Moraxella, plasmid-encoded enzymes are also present, which was previously unknown to our knowledge[20], and indicates that the ability to convert DAG to TG can likely be shared between bacteria and across different taxonomic families.

Supplementary Table 1. General statistics per sample including sample_type, sample_set (train or test), number of contigs per subset (Prokaryotes, Eukaryotes, plasmids, viruses and total), number of contigs in bins (for Prokaryotes and Eukaryotes), number of filtered reads (MG and MT), number of peptides, numbered of mapped reads (MG and MT), number of ORFs, transcripts and proteins as well as number of KOs in the ORFs, transcripts and proteins.

Supplementary Table 2. Number of representative MAGs (rMAGs), contigs (rContigs) and ORFs per biological subset (prokaryotic, eukaryotic, plasmidial and viral).

Supplementary Table 3. Reported p-values for the Shapiro test performed on 10 random subsets (with 5000 data points each) of the betas from the batch-effect correction of the MG ORF data. The two technical variables are the number of reads and the average read length per sample.

Supplementary Table 4. Reported p-values for the Shapiro test performed on 10 random subsets (with 5000 data points each) of the betas from the batch-effect correction of the MT ORF data. The two technical variables are the number of reads and the average read length per sample.

Supplementary Table 5. Environmental parameters manually collected by the researchers at the sampling site.

Supplementary Table 6. Environmental parameters automatically collected by the sensors of the WWTP.

Supplementary Table 7. Exact p values for the tests in **Figure 3**.

Bibliography

1. Hand, D. J. & Vinciotti, V. Local Versus Global Models for Classification Problems. *Am. Stat.* (2003) doi:10.1198/0003130031423.

2. Abanda, A., Mori, U. & Lozano, J. A. A review on distance based time series classification. *Data Min. Knowl. Discov.* (2019) doi:10.1007/s10618-018-0596-4.

3. Arul, M. & Kareem, A. Applications of shapelet transform to time series classification of earthquake, wind and wave data. *Eng. Struct.* (2020) doi:10.1016/j.engstruct.2020.111564.

4. Keogh, E., Chu, S., Hart, D. & Pazzani, M. SEGMENTING TIME SERIES: A SURVEY AND NOVEL APPROACH. in 1–21 (2004). doi:10.1142/9789812565402_0001.

5. Kunath, B. J. *et al.* From proteins to polysaccharides: lifestyle and genetic evolution of Coprothermobacter proteolyticus. *ISME J.* (2019) doi:10.1038/s41396-018-0290-y.

6. Zhang, Z. *et al.* Dynamic Time Warping under limited warping path length. *Inf. Sci. (Ny).* (2017) doi:10.1016/j.ins.2017.02.018.

7. Petropoulos, F. *et al.* Forecasting: theory and practice. *Int. J. Forecast.* (2020) doi:10.1016/j.ijforecast.2021.11.001.

8. Gilmour, S., Degenhardt, L., Hall, W. & Day, C. Using intervention time series analyses to assess the effects of imperfectly identifiable natural events: A general method and example. *BMC Med. Res. Methodol.* (2006) doi:10.1186/1471-2288-6-16.

9. Alter, O., Brown, P. O. & Botstein, D. Singular value decomposition for genome-Wide expression data processing and modeling. *Proc. Natl. Acad. Sci. U. S. A.* (2000) doi:10.1073/pnas.97.18.10101.

10. Azaele, S., Pigolotti, S., Banavar, J. R. & Maritan, A. Dynamical evolution of ecosystems. *Nature* **444**, 926–928 (2006).

11. Ives, A. R. & Carpenter, S. R. Stability and diversity of ecosystems. *Science* (2007) doi:10.1126/science.1133258.

12. Tyson, G. W. *et al.* Community structure and metabolism through reconstruction of microbial genomes from the environment. *Nature* **428**, 37–43 (2004).

13. Venter, J. C. *et al.* Environmental Genome Shotgun Sequencing of the Sargasso Sea. *Science (80-.).* (2004) doi:10.1126/science.1093857.

14. Poretsky, R. S. *et al.* Analysis of Microbial Gene Transcripts in Environmental Samples. *Appl. Environ. Microbiol.* **71**, 4121–4126 (2005).

15. Wilmes, P. & Bond, P. L. The application of two-dimensional polyacrylamide gel electrophoresis and downstream analyses to a mixed community of prokaryotic microorganisms. *Environ. Microbiol.* **6**, 911–920 (2004).

16. Osaka, T., Ebie, Y., Tsuneda, S. & Inamori, Y. Identification of the bacterial community involved in methane-dependent denitrification in activated sludge using DNA stable-isotope probing. *FEMS Microbiol. Ecol.* (2008) doi:10.1111/j.1574-6941.2008.00473.x.

17. Fang, W. *et al.* Organic carbon and eukaryotic predation synergistically change resistance and resilience of aquatic microbial communities. *Sci. Total Environ.* **830**, 154386 (2022).

18. Chen, G., Harwood, J. L., Lemieux, M. J., Stone, S. J. & Weselake, R. J. Acyl-CoA:diacylglycerol acyltransferase: Properties, physiological roles, metabolic engineering and intentional control. *Prog. Lipid Res.* **88**, 101181 (2022).

19. Wang, Y. *et al.* Successional dynamics and alternative stable states in a saline activated sludge microbial community over 9 years. *Microbiome* **9**, 199 (2021).

20. Arabolaza, A., Rodriguez, E., Altabe, S., Alvarez, H. & Gramajo, H. Multiple Pathways for Triacylglycerol Biosynthesis in Streptomyces coelicolor. *Appl. Environ. Microbiol.* **74**, 2573–2582 (2008).
