# Supplementary material for: Forecasting the dynamics of a complex microbial community using integrated meta-omics
Source: Nat Ecol Evol. 2023 Nov 13;8(1):32–44. doi: 10.1038/s41559-023-02241-3 (PMC10781640; doi:10.1038/s41559-023-02241-3)
Supplement: Supplementary file 2 — Reporting Summary [file 41559_2023_2241_MOESM2_ESM.pdf]

nature portfolio

Corresponding author(s): Paul Wilmes
Francesco Delogu

Last updated by author(s): Sep 15, 2023

Reporting Summary

Nature Portfolio wishes to improve the reproducibility of the work that we publish. This form provides structure for consistency and transparency in reporting. For further information on Nature Portfolio policies, see our Editorial Policies and the Editorial Policy Checklist.

Statistics

For all statistical analyses, confirm that the following items are present in the figure legend, table legend, main text, or Methods section.

n/a	Confirmed	
☐	☒	The exact sample size (*n*) for each experimental group/condition, given as a discrete number and unit of measurement
☐	☒	A statement on whether measurements were taken from distinct samples or whether the same sample was measured repeatedly
☐	☒	The statistical test(s) used AND whether they are one- or two-sided<br>*Only common tests should be described solely by name; describe more complex techniques in the Methods section.*
☐	☒	A description of all covariates tested
☐	☒	A description of any assumptions or corrections, such as tests of normality and adjustment for multiple comparisons
☐	☒	A full description of the statistical parameters including central tendency (e.g. means) or other basic estimates (e.g. regression coefficient) AND variation (e.g. standard deviation) or associated estimates of uncertainty (e.g. confidence intervals)
☐	☒	For null hypothesis testing, the test statistic (e.g. *F*, *t*, *r*) with confidence intervals, effect sizes, degrees of freedom and *P* value noted<br>*Give P values as exact values whenever suitable.*
☒	☐	For Bayesian analysis, information on the choice of priors and Markov chain Monte Carlo settings
☒	☐	For hierarchical and complex designs, identification of the appropriate level for tests and full reporting of outcomes
☐	☒	Estimates of effect sizes (e.g. Cohen's *d*, Pearson's *r*), indicating how they were calculated

Our web collection on statistics for biologists contains articles on many of the points above.

Software and code

Policy information about availability of computer code

Data collection	Sequencing data were generated with an illumina machine and the software provided by the manufacturer for the base calling.
Data analysis	The software (and versions when available) used within this work include:<br>SPAdes (ver. 3.13.1)<br>cbar (ver. 1.2)<br>plasflow (ver. 1.1.0)<br>VirSorter (ver. 1.0.6)<br>deepVirFinder (ver 1.0)<br>euktep (ver. 0.6.7)<br>metaeuk (ver. 3.8dc7e0b)<br>IMP (ver. 3)<br>dRep (ver. 0.5.4)<br>CheckM (ver. 1.0.7)<br>CD-HIT (ver. 4.6.8)<br>mmseq (ver. 1.0.9)<br>CAT/BAT (ver. 5.1.2)<br>mantis (ver. 1.02)<br>AUGUSTUS (ver. 3.3.3)<br>R statistical package (ver. 3.4.4)<br>Cytoscape (ver 3.5.1)<br>snakemake (ver from 7.7.0)

bwa (ver 0.7.17)
bam2hits (ver 1.0.9)
mmseq (ver 1.0.9)
mmcollapse
SearchGUI(ver 3.3.20)
PeptideShaker (ver1.16.45)
samtools (ver 1.11)
R (ver 3.3.4)
fable (ver 0.3.1)
lmtest (ver 0.9-38)
rEDM (ver 1.14.0)

For manuscripts utilizing custom algorithms or software that are central to the research but not yet described in published literature, software must be made available to editors and reviewers. We strongly encourage code deposition in a community repository (e.g. GitHub). See the Nature Portfolio guidelines for submitting code & software for further information.

Data

Policy information about availability of data

All manuscripts must include a data availability statement. This statement should provide the following information, where applicable:

- Accession codes, unique identifiers, or web links for publicly available datasets
- A description of any restrictions on data availability
- For clinical datasets or third party data, please ensure that the statement adheres to our policy

The generated metagenomics and metatranscriptomics reads (FASTQ) files, as well as the previously produced data, are available as NCBI BioProject PRJNA230567. The MP data from the PRIDE repository with accession number PXD013655. The environmental parameter measurements are available at https://github.com/fdelogu/microforecast.

Research involving human participants, their data, or biological material

Policy information about studies with human participants or human data. See also policy information about sex, gender (identity/presentation), and sexual orientation and race, ethnicity and racism.

Reporting on sex and gender	N/A
Reporting on race, ethnicity, or other socially relevant groupings	N/A
Population characteristics	N/A
Recruitment	N/A
Ethics oversight	N/A

Note that full information on the approval of the study protocol must also be provided in the manuscript.

Field-specific reporting

Please select the one below that is the best fit for your research. If you are not sure, read the appropriate sections before making your selection.

☐ Life sciences ☐ Behavioural & social sciences ☒ Ecological, evolutionary & environmental sciences

For a reference copy of the document with all sections, see nature.com/documents/nr-reporting-summary-flat.pdf

Ecological, evolutionary & environmental sciences study design

All studies must disclose on these points even when the disclosure is negative.

Study description	Analysis and forecasting of a multi meta-omics time series from the foam community resident in the anoxic tank from a wastewater treatment plant.
Research sample	The samples are individual floating foam islets (one per sample) from the anoxic tank of the biological wastewater treatment plant in Schifflange (Luxembourg).
Sampling strategy	Individual floating sludge islets were collected from the same spot of the anoxic tank, along with physico-chemical parameters of the water, i.e. pH, temperature, conductivity, oxygen.<br>This work is part of an ongoing multi-annual project. Thus, all the samples were subjected to the same experimental protocols. Please refer to detailed methods on sampling procedures in previous publications:

https://doi.org/10.1038/ncomms6603
https://doi.org/10.1038/npjbiofilms.2015.7

Data collection	Laura A. Lebrun and Emilie E.L. Muller performed the concomitant biomolecular extractions resulting in fractions of DNA, RNA, proteins and metabolites for each in situ sample. R. Halder performed the DNA and RNA library preparation and next-generation sequencing (NGS) to obtain MG and MT data.
Timing and spatial scale	All the samples were taken always from the same tank. The 52 training set covers the time period March 2011 to May 2012 with roughly weekly sample. The test set includes 21 samples from the month of June in the years 2012-2016.
Data exclusions	No sample was excluded. ORFs, transcripts or proteins detected in less than 20% of the training set were not analysed.
Reproducibility	Experimental procedures adhered to previously published protocols. Open source software was used in all the computational analyses. The code for the statistical analysis is available on Github.
Randomization	The training and test set samples were extracted and sequenced in different time and using different methods as specified in the manuscript making a full randomization impossible. However, the batch effect has been formally addressed. The training set series was randomized for extraction and sequencing.
Blinding	Blinding does not apply.

Did the study involve field work? ☒ Yes ☐ No

Field work, collection and transport

Field conditions	The anoxic tank of the biological wastewater plant was sampled under any condition (as long as there was foam present).
Location	SIVEC wastewater treatment plant in Schifflange (Luxembourg).
Access & import/export	Access was granted to the research personnel based on agreement between the principal investigator, Prof. Paul Wilmes (on behalf of the research institution), and the wastewater treatment facility management (Mr. Bissen and Mr. Di Pentima) from the Syndicat Intercommunal a Vocation Ecologique (SIVEC), Schifflange, Luxembourg. All research personnel are informally introduced to the management and personnel of the facility prior to conducting any work. Research personnel were not provided with keys or electronic access cards, and thus could only enter the premises upon the permission of personnel at the entrance of the facility.
Disturbance	Sampling had a minimum-to-no impact on the operations of the wastewater treatment facility. The work of the researchers did not require (complete or partial) shutdown or any operational disruption of the facility. Sampling was performed by the research personnel (Emilie E.L. Muller and Laura A. Lebrun) without any involvement of the staff of the facility. Research personnel either brought their own equipment or used equipment from the site, which was dedicated to them, thus not hindering any operations or personnel within facility. Researchers could access operational readings (e.g. temperature, inflow, outflow, etc.) of the facility directly via a dedicated web portal of the facility using login credentials provided by the facility management. Two formal meetings weres organized between researchers and management of the facility over the past five years.

Reporting for specific materials, systems and methods

We require information from authors about some types of materials, experimental systems and methods used in many studies. Here, indicate whether each material, system or method listed is relevant to your study. If you are not sure if a list item applies to your research, read the appropriate section before selecting a response.

Materials & experimental systems

n/a	Involved in the study	
☒	☐	Antibodies
☒	☐	Eukaryotic cell lines
☒	☐	Palaeontology and archaeology
☒	☐	Animals and other organisms
☒	☐	Clinical data
☒	☐	Dual use research of concern
☒	☐	Plants

Methods

n/a	Involved in the study	
☒	☐	ChIP-seq
☒	☐	Flow cytometry
☒	☐	MRI-based neuroimaging
